# Trajectory of suicidal ideation among medical students during the COVID-19 pandemic: the role of childhood trauma

**DOI:** 10.1186/s12888-023-04582-6

**Published:** 2023-02-06

**Authors:** Jiang Nan, Nen Salina, Sheau Tsuey Chong, Hong-juan Jiang

**Affiliations:** 1 Centre for Research in Psychology and Human Well-Being, Faculty of Social Sciences and Humanities, Universiti Kebangsaan Malaysia, 43600 Bangi, Malaysia; 2 Hainan Medical University, Haikou, 571199 China; 3 Counselling Program, Postgraduate Secretariat, Faculty of Social Sciences and Humanities, Universiti Kebangsaan Malaysia, 43600 Bangi, Malaysia; 4 The First Affiliated Hospital of Hainan Medical University, Haikou, 571199 China

**Keywords:** Suicidal ideation, Childhood trauma, GMM, COVID-19

## Abstract

**Background:**

The aim of this study was to understand the longitudinal trajectory of suicidal ideation (SI) among Chinese medical students and the role of childhood trauma (CT).

**Methods:**

Using a whole-group sampling method, we assessed SI in 2192 (male = 834, female = 1358) medical students on three occasions over a period of one year. The Suicidal Ideation Self-Assessment Scale (SISAS) and the Childhood Trauma Questionnaire-Short Form (CTQ-SF) were used to assess SI and CT. The growth mixture modeling (GMM) was used to classify the developmental trajectory of SI.

**Results:**

A greater number of medical students were experiencing suicidal ideation during the COVID-19 pandemic. The trajectory of SI among medical students was divided into two groups: a low risk, slowly rising group and a high risk, continuous group. The low risk, slowly rising group had a significant time effect (B = 1.57, *p* < 0.001) and showed a slowly increasing trend. Emotional neglect (EN), physical neglect (PN), emotional abuse (EA) and physical abuse (PA) all had significant positive predictive effects for the high risk, continuous group (B = 0.18–0.65, *P* < 0.01).

**Conclusion:**

The trajectory of SI among medical students can be divided into a low risk, slowly rising group and a high risk, continuous group; the more EN, PN, EA and PA experienced during childhood, the more likely medical students are to develop a high risk, continuous state of SI.

## Background

Suicide is a serious global public health problem. More than 700,000 people worldwide commit suicide each year, and suicide is the fourth leading cause of death among people aged 15–29 years [1]. The process of suicide in individuals generally goes through three stages: developing suicidal ideation (SI), making a suicide plan, and committing suicidal acts [2]. According to the interpersonal theory of suicide, an individual will only commit suicide if he or she has the following: a sense of unmet belonging, a sense of perceived burdensomeness and the ability to commit suicide. Additionally, he or she is highly vulnerable to SI when both unmet belonging and perceived burdensomeness are present, but both are not stable traits and are susceptible to interpersonal factors and the individual's early experiences [3]. Suicidal ideation is an important predictor of suicidal behavior. Suicidal ideation may not lead to suicidal behavior, but it provides an opportunity for individuals to re-examine the meaning of life [2, 4]. Therefore, it is important to understand the characteristics of SI and to develop targeted prevention and intervention approaches to reduce the occurrence of SI.

Globally, the college student population has high levels of SI, planning, and attempts. In a survey of the prevalence of suicidal behaviour and psychological distress among university students in 12 countries, 29% of the sample reported having considered suicide and 7% reported attempting suicide, with both non-fatal suicidal behaviour and psychological distress being frequent events among university students and showing considerable variation [5] A meta-analysis found that the pooled prevalence of suicide attempts among Chinese university students was 2.8%, much higher than that reported in the general Chinese population [6]. As college students move from adolescence into early adulthood, they are faced with multiple pressures from performing in academics, developing a sense of self-identity, maintaining interpersonal relationships, and planning for a career. Medical students (being awarded a medical degree) are a special group with a higher prevalence of mental health problems than students from other specialty [7]. Long hours of study, heavy academic loads, competition with peers and other stressors can affect the mental health of medical students [8, 9] and lead to high levels of SI [10]. The common view is that SI is a fleeting thought; however, longitudinal findings found that 16% of those who reported SI at baseline levels still reported SI at follow-up 9 years later, meaning that there is a persistent effect of SI [11]. The same results were confirmed in the adolescent population [12]. Several studies have examined the SI according to longitudinal experimental design. For example, Chen and colleagues [13] observed that the SI in high school students fluctuates widely in 21 months (the students completed the questionnaire of SI every 3 months). Xiao and colleagues [14] presented that there were three types of trajectories of SI in adolescents: low level-stable, high level-declining, and a moderate level that is either declining or rising. Another follow-up study showed no change in the overall level of SI among Chinese adolescents [15]. There is no research on the development of SI among medical students, so is there a unique trajectory of SI among this unique student population?

The cumulative situational risk model suggests that the more negative situations an individual experiences early in life, the higher the likelihood of cumulative or superimposed developmental risk and the greater the risk of SI [16]. Among the negative situations that lead to SI, childhood trauma (CT) is a significant predictor. CT is defined as actual or potential harm to a child's health, survival, development or self-esteem by the child's guardian or someone the child trusts and includes various forms of emotional abuse (EA), physical abuse (PA), sexual abuse (SA), emotional neglect (EN), and physical neglect (PN) [17]. Individuals who have been abused in childhood have a high frequency of suicidal thinking and suicide attempts, and will encounter more difficulties in the subsequent development tasks, showing more depression, SI and suicide attempts [18–20]. Although most studies confirm the strong relationship between childhood abuse and SI and suicidal behavior, some studies have found that only childhood sexual abuse directly affects SI [21]. It has also been shown that CT, such as physical and emotional abuse, does not effectively predict SI [22]and that some individuals maltreated in childhood have relatively lower SI [23, 24]. Based on previous research, the relationship between CT and SI is uncertain, but CT has a cumulative negative impact and is associated with the development of negative emotions and behaviors in individuals. This study therefore seeks to understand whether different forms of CT over time affect changes in SI.

Between January 2020 and December 2020, China's fight against the new crown epidemic went through three phases: an outbreak period (a sharp increase in the number of infections, home quarantine of citizens, and online courses for students.), a period of de-escalation (around April 2020, when the national outbreak is largely under control, with no new cases in Hubei province and a decline in new cases nationwide), and normalised prevention and control (sporadic distribution of cases, a shift from emergency to normalisation, citizens can move across provinces with protection, and students begin face-to-face teaching). Emerging infectious diseases have been shown to increase the incidence rate of suicide during outbreaks or pandemics; the 1918 influenza in the United States, the 2003 severe acute respiratory syndrome (SARS) pandemic, and the 2014 Ebola outbreak in West Africa all reported an increase in suicidal ideation and an increase in the number of deaths by suicide [25–27]. Perceived threats to life, social isolation, and food and resource insecurity expose the entire population to trauma, and the impact on public mental health status is widespread and long-lasting [28, 29]. COVID-19, the most severe pandemic since Spanish influenza, spread rapidly across the globe, and strict control measures such as isolation, lockdown, physical distance, and online learning were adopted by various countries and were believed to be effective in controlling the spread of the virus. However, mass isolation can lead to individuals experiencing a frustrated sense of belonging [30], where individuals feel a lack of connection with others and are unable to receive support and help from others. On the other hand COVID-19 leads to unemployment, the inability to provide for loved ones and the experience that one may become a burden to others, i.e. people perceive themselves as burdensome, which can increase their suicidal thoughts [31, 32]. Frustrated belonging and perceived burdensomeness are two separate and interrelated constructs that together lead to suicidal ideation [32]. The current study found that the prevalence of suicidal ideation was elevated in the early years of the pandemic, with 18–24 year olds being the highest group for suicidal ideation [33–35]. Although many studies have confirmed that the COVID-19 epidemic affects SI among college students, the impact on SI among Chinese medical students is elusive. COVID-19 is a socially traumatic public health event, especially for individuals who have experienced a traumatic childhood event and are more vulnerable to the effects of COVID-19 [36]. Several studies have found that individuals who experienced adverse childhood events during the COVID-19 pandemic exhibited higher levels of anxiety, depression and PTSD symptoms [37–39].In addition, individuals with a history of childhood abuse experienced increased psychological distress and PTSD symptoms during the pandemic, even over and above demographic characteristics and stressors associated with COVID-19 [40]. This may suggest that childhood trauma may be a risk factor for psychological distress during COVID-19.

In summary, most of the existing studies on SI are cross-sectional surveys, which do not well reflect developmental characteristics and trends in SI. The few longitudinal studies on SI have only described simple outcomes and have not examined trends and changes in SI, nor have they explored group heterogeneity. While studies on factors influencing SI generally support the association of gender, place of origin, major, and CT with SI, there are a few studies with different results, and the use of longitudinal follow-up studies and exploration of heterogeneity would be more effective in clarifying the relationship of these studies to the mainstream. Therefore, this study uses growth mixture modeling with medical students to first explore the trajectory of SI among college students and whether there are heterogeneous latent category differences and second, to further understand the predictive effects of gender, place of birth, major, and CT on the trajectory of SI among college students. In this study, we aim to enrich existing theories and provide references for the prevention of suicide among medical students.

## Methods

### Participants

This study was reviewed by the Ethics Committee of Hainan Medical College (HYLL2021234). All participants were medical students from Hainan Province in China and signed the informed consent form to voluntarily participate in this study. The survey was conducted using an online questionnaire and lasted for 1 year, three times. The three surveys were conducted: T1: 1 December – 10 December 2019; T2: 1 June – 10 June 2020; T3: 1 December – 10 December 2020. Among the 2713 participants included at T1, 2560 (94.3%) were followed at T2 and 2192 (80.8%) completed the full 1-year follow-up (T3).

The demographic information of each participant, including gender, age, and major was collected. Based on the guiding principles of emergent psychological crisis intervention in COVID-19 [41], the populations affected by COVID-19 were divided into the following four levels: level 1, patients with severe symptoms of COVID-19, front-line medical workers, CDC researchers, or administrative staff; level 2, patients with mild symptoms of COVID-19, close contacts, suspected COVID-19 patients, or patients with fever who were admitted to the hospital for treatment; level 3, individuals related to the level 1 and level 2 group members, such as family members, colleagues, friends, and rescuers, including commanders, administrative staff, and volunteers; level 4, residents of affected areas, susceptible groups, and the general public. In addition, the participants were asked whether they had a family member or friend suspected of having COVID-19, and whether they had family or friends being healthcare workers.

### Measures

#### Suicidal Ideation Self-Assessment Scale (SISAS)

The scale developed by Xia Chaoyun [42] was used to assess individuals' SI and intensity over the past 12 months. A total of 26 items, including four subscales of despair, optimism, sleep, and masking, were scored on a "yes = 1" and "no = 0" scale. The measure was unreliable if the masking subscale score was ≥ 4. SI was considered to be presented if the total score on the Despair, Optimism and Sleep subscales was ≥ 12 and the score on the Masking subscale was ≤ 4. The higher the score, the stronger the SI. The scale has good reliability and validity among Chinese university students. The Cronbach's alpha coefficients for the three measures in this study were 0.81, 0.87 and 0.83, respectively.

#### Childhood Trauma Questionnaire-Short Form (CTQ-SF)

This is used to assess traumatic experiences in childhood. There are 28 items, including 5 subscales of emotional abuse, physical abuse, sexual abuse, emotional neglect, and physical neglect (25 items in total) and a validity scale (3 items in total), scored on a scale of 1 (never)—5 (always), with higher scores indicating more severe childhood abuse. The validity scale is scored on a two-point scale of 0–1, and a total validity score of greater than 3 invalidates the questionnaire. The CTQ-SF has high reliability and validity among Chinese undergraduates [43]. The criteria for determining trauma in childhood in this scale were: total physical abuse score ≥ 10, total physical neglect score ≥ 10, total emotional abuse score ≥ 13, total emotional neglect score ≥ 15, and total sexual abuse score ≥ 8 [44]. The reliability of the scale is good, with a Cronbach's alpha coefficient of 0.81 in this study.

### Statistical analysis

Statistical analysis was conducted using IBM SPSS Version 22.0, and Mplus 7.0 was used for Growth Mixture Modelling (GMM) analysis, which can accurately distinguish the differences between individuals while analysis the trajectory of SI, providing an empirical basis and theoretical reference for the identification of SI and planned interventions. The data analysis was conducted in three steps.

In the first step, using SPSS, t tests and Chi-square tests, with Bonferroni corrections for multiple tests, were employed to reveal whether the variables of participants who completed the three assessments (T1-T3) were significantly different from the variables of those who did not complete the three assessments. Spearman’s rho and Kendall’s tau coefficient were used to determine the degree of correlation between variables.

In the second step, Latent category model fit was assessed to analysis whether there were heterogeneous latent category differences in the trajectories of change in SI among university students. The indicators and criteria used in this study to evaluate the model fit were as follows: (1) Akaike information criterion (AIC) and Bayesian information criterion (BIC) in which the smaller the value is, the better; (2) entropy, which has a value approximately equal to 0.8, indicates that the classification accuracy exceeds 90%, and is more accurate the closer it is to 1; (3) the Likelihood Ratio Test (Lo-Mendell-Rubin, LMR) and Bootstrapped Likelihood Ratio Test (BLRT), which were used to determine if the model for the current category is significantly better than the model for the previous category, as judged by the significance of the p value (the number of categories starts from 1 and is set in increasing order).

In the third step, the effects of the covariates age, gender, major and CT on the potential trajectories of change in SI among medical students were tested. Based on the potential categories of the trajectory of change in suicidal risk obtained in step 2, an unordered multivariate logistic regression model was developed using the classification of potential categories as the dependent variable and gender, major and CT as predictor covariates.

## Results

### Demographic and psychometric characteristics

The demographics, SI, and CT in the total sample are presented in Table 1. The 2192 participants in this study ranged in age from 16 to 23 and were on average 18 years old. There were 38.0% males and 62.0% females; 57.1% were from urban areas while 42.9% were from rural areas; 47.7% majored in clinical medicine while 52.3% majored in other specialties, such as nursing, psychiatry, medical imaging, and so on; all of them received medical degrees. Additionally, 97.6% of the respondents reported not having exposure to COVID-19, 69.5% reported not having family or friends who worked in healthcare, and 96.5% reported not having been infected with COVID-19.

**Table 1:** The demographics, suicidal ideation, and childhood trauma in total sample

Variable	Total (*n* = 2192)	Low risk, slowly rising SI group	high risk, continuous SI group
		**(*n* = 1853, 84.5%)**	**(*n* = 339, 15.5%)**
**Age(range), years ^**	18.46** ± **0.86 (16–23)	18.44** ± **0.86 (16–23)	18.57** ± **0.90 (16–22)
**Gender**			
Male	834 (38.0)	704 (38.0)	130 (38.3)
Female	1358 (62.0)	1149 (62.0)	209 (61.7)
**Birthplace**			
Urban	1251 (57.1)	1049 (56.6)	202 (59.6)
Rural	941 (42.9)	804 (43.4)	137 (40.4)
**Major**			
Clinical Medicine	1045 (47.7)	874 (47.2)	171 (50.4)
Nursing	417 (19.0)	356 (19.2)	61 (18.0)
Medical Laboratory Science	170 (7.8)	146 (7.9)	24 (7.1)
Stomatology	69 (3.1)	57 (3.1)	12 (3.5)
Psychiatry	31 (1.4)	26 (1.4)	5 (1.5)
Pharmacy	174 (8.0)	151 (8.2)	23 (6.8)
Public Health & Preventive Medicine	47 (2.1)	41 (2.2)	6 (1.8)
Medical Imaging	55 (2.5)	43 (2.3)	12 (3.5)
Traditional Chinese Medicine	184 (8.4)	159 (8.5)	25 (7.4)
**COVID-19 exposure**			
Level 1	0	0	0
Level 2	0	0	0
Level 3	54 (2.4)	38 (2.1)	16 (4.7)
Level 4	2138 (97.6)	1815 (97.9)	323 (95.3)
**Whether the participant’s family or friends are healthcare workers**			
Yes	668 (30.5)	546 (29.5)	122 (36.0)
No	1524 (69.5)	1307 (70.5)	217 (64.0)
**Whether the participant’s family or friends had been infected with COVID-19**			
Someone diagnosed	0	0	0
Someone suspected	0	0	0
No infection	2115 (96.5)	1790 (96.6)	324 (95.6)
Unclear	77 (3.5)	63 (3.4)	15 (4.4)
**Suicidal ideation T1^**	4.26 (3.54)	3.18 (2.31)	10.18 (3.27)
**Suicidal ideation T2^**	7.40 (2.85)	6.79 (2.10)	10.74 (3.93)
**Suicidal ideation T3^**	6.64 (3.21)	6.30 (2.87)	8.50 (4.20)
**Emotional Neglect^**	8.45 (4.02)	7.97 (3.71)	11.12 (4.58)
**Physical Neglect^**	6.88 (2.55)	6.62 (2.33)	8.32 (3.16)
**Emotional Abuse^**	7.28 (2.51)	6.91 (2.16)	9.31 (3.20)
**Physical Abuse^**	6.01 (2.08)	5.90 (2.00)	6.63 (2.38)
**Sexual Abuse^**	5.64 (1.82)	5.60 (1.83)	5.88 (1.76)

Note: ^data were expressed as mean (SD), others, n (%)

There were no differences between the participants who completed the three assessments (T1-T3) and those who did not. The SI scores of medical school students in this study were 4.26 ± 3.54, 7.40 ± 2.85, and 6.64 ± 3.21 at T1, T2, and T3 (see Table 1), which were significantly different from the result (5.02 ± 3.55) of Xia Zhaoyun [42] (*t* = -9.65, 38.98, and 23.63, all *p* < 0.001). The prevalence of SI fluctuates in three surveys, 3.9% at T1, 5.2% at T2, and 5.1% at T3, with an overall increase. lower than the 5.4% in the Xia Zhaoyun [42] study, according to the scale's delineation scores. In addition, 825 (30.4%) participants reported one or more childhood trauma experiences, including 188 (6.9%) of emotional neglect, 295 (10.9%) of physical neglect, 97 (3.6%) of emotional abuse, 132 (4.9%) of physical abuse and 113 (4.2%) of sexual abuse.

Zero-order correlations of dichotomous and continuous background variables, SI (at T1-T3), and subscales (EN, PN, EA, PA, SA) of CT are presented in Table 2. Age was positively correlated with SI (at T2) (*r* = 0.05, *p* < 0.05), gender was negatively correlated with SI (at T1-T3) (*r* = -0.05, *p* < 0.05; *r* = -0.05, *p* < 0.05; *r* = -0.07, *p* < 0.001), Major and SI (at T1, T2) were negatively correlated (both *r* = -0.05, *p* < 0.05); EN, PN, and EA were positively correlated with SI (at T1, T2),, PA was positively correlated with SI (at T1,T2,T3),and SA was positively correlated with SI (at T1) (see Table 2 for details).

**Table 2:** Correlations of SI (at baseline, 6-month and 12-month follow-up), childhood trauma at baseline and Demographics

**Variable**	T1-SI	T2-SI	T3-SI	EN	PN	EA	PA	SA	Age	gender	birthplace	major	a1	a2	a3
T1-SI	1														
T2-SI	0.38^***^	1													
T3-SI	0.28^***^	0.34^***^	1												
EN	0.29^***^	0.11^***^	0.04	1											
PN	0.25^***^	0.08^***^	0.02	0.62^***^	1										
EA	0.17^***^	0.06^***^	0.02	0.31^***^	0.42^***^	1									
PA	0.37^***^	0.16^***^	0.11^***^	0.38^***^	0.44^***^	0.58^***^	1								
SA	0.08^***^	0.01	-0.02	0.21^***^	0.39^***^	0.53^***^	0.48^***^	1							
Age	0.03	0.05^*^	-0.01	0.02	0.05^*^	0.01	0.01	0.05^*^	1						
gender	-0.05^*^	-0.05^*^	-0.07***	0.04	0.08^***^	0.15^***^	0.02	0.13^***^	-0.01	1					
birthplace	-0.14	-0.01	-0.01	0.02	-0.04*	0.02	0.02	-0.04	-0.07**	0.01	1				
major	-0.05^*^	-0.05^*^	0.01	0.03	-0.03	-0.05^*^	-0.03	-0.04	-0.10^*^	0.02	0.15^***^	1			
a1	0.01	-0.04	0.02	0.01	0.01	0.01	-0.02	0.03	-0.01	-0.04	-0.01	0.01	1		
a2	0	0	0.02	0.01	0.03	0.01	0.01	0.03	0.01	0.02	0.02	0.01	0.03	1	
a3	-0.01	-0.02	-0.01	0.01	0.03	0.01	-0.01	0.02	0.04	-0.05*	-0.02	0.02	0.11^***^	-0.24***	1

Note: *SI* Suicidal ideation, *EN* Emotional Neglect, *PN* Physical Neglect, *EA* Emotional Abuse, *PA* Physical Abuse, *SA* Sexual Abuse, *T1* baseline, *T2* 6-month follow-up, *T3* 12-month follow-up; Gender (1 = male, 2 = female); Birthplace (1 = urban, 2 = rural); Major (1 = Clinical Medicine, 2 = Non-Clinical Medicine); a1: Type of COVID-19 exposure (1 = Level 1, 2 = Level 2, 3 = Level 3, 4 = Level 4); a2: COVID-19 infection of a family member or friend (1 = Someone diagnosed, 2 = Someone suspected, 3 = No infection, 4 = Unclear); a3: family or friends of the participants being healthcare workers (1 = Yes, 2 = No). Spearman’s rho was used to analyze the correlation between SI (T1, T2, T3), CT (EN, PN, EA, PA, SA), and Age. Kendall’s tau coefficient was used to analyze the correlation between Demographics and SI (T1, T2, T3), CT (EN, PN, EA, PA, SA)

^*^P < 0.05

^**^P < 0.01

^***^P < 0.001

### Results of the growth mixture modeling

#### SI trajectory groups

As seen in Table 3, the information indices AIC, BIC, and aBIC continue to decrease as the number of categories increases, with the highest entropy when two categories are retained. The LMR and BLRT values reached significance levels when both categories were retained, and on balance, retaining 2 categories was the optimal classification model. The results of two categories of demographics and childhood trauma are presented in Table 1.

**Table 3:** The latent growth mixture model analysis indicators of suicide ideation

category	*K*	Log(L)	AIC	BIC	aBIC	Entropy	LMR	BLRT	category probability
1	8	-17025.69	34067.39	34112.93	34087.51	-	-	-	-
**2**	**27**	**-16687.26**	**33428.53**	**33582.23**	**33496.45**	**0.83**	** < 0.001**	** < 0.001**	**84.5/15.5**
3	46	-16580.10	33252.20	33514.06	33367.91	0.65	0.594	0.595	46.1/43.5/10.4

Note: Final solutions are in bold. *AIC* Akaike information criterion, *BIC* Bayesian information criterion, *VLMR* Vuong-Lo-Mendell-Rubin test, *BLRT* bootstrap likelihood ratio test

Analysis of the fitted information indices for the mixed growth model revealed that a division into 2 potential categories was most appropriate. We will therefore build on this to further examine the trajectory of each latent class. The mean values of the intercept (α) and slope (B) are available in the mixed model.

The intercept (α) describes the average initial state of the individuals, while the mean of the slope (B) describes the average growth rate between time points. As the results in Table 4 show, the mean values of the intercept (α) for each latent class were C1: 4.00 (SE = 0.12, t = 31.32, *P* < 0.001) and C2: 10.32 (SE = 0.33, t = 31.06, *P* < 0.001). There were significant differences in the mean intercept values between each latent class, with C2 having a higher initial value and C1 having a lower value.

**Table 4:** The mean intercept values of the latent class

category	Estimate	*S.E*	Est./*S.E*	***P***
C1	4	0.12	31.32	< 0.001
C2	10.32	0.33	31.06	< 0.001

Note: C1: “low risk, slowly rising” SI group; C2: “high risk, continuous” SI group

The average growth rate of each latent class was described by the mean of the slopes (B) of the potential categories; the results in Table 5 show that the means of the slopes (B) of the 2 potential categories were C1: 1.57 (SE = 0.12, t = 12.57, *p* < 0.001) and C2: -0.68 (SE = 0.41, t = 31.32, *p* = 0.098). The slope of group C1 is a significantly different, smaller positive number, indicating that the level of SI in group C1 increased significantly over time. The slope of group C2 was negative, and the absolute value was small, indicating that the level of SI in group C2 showed a decreasing trend over time, but the difference in the level of SI between the three time points was not significant.

**Table 5:** The mean slope values of the latent class

category	Estimate	*S.E*	Est./*S.E*	***P***
C1	1.57	0.12	12.57	< 0.001
C2	-0.68	0.41	-1.65	0.098

Note: C1: “low risk, slowly rising” SI group; C2: “high risk, continuous” SI group

Combining the results of the intercept (α) mean with the slope (B) mean, the trajectory characteristics of the 2 potential categories of SI groups can be derived. The C1 group showed a low to high change in the level of SI, but the difference in the level of SI between the three time points was low, so the C1 group could be named “low risk, slowly rising group” (84.5% of the sample), whereas the C2 group showed a high to low change in the level of SI, but the change was not significant. Therefore, the C2 group can be named “high risk, continuous group” (15.5% of the sample).

### Analysis of factors influencing SI in latent class groups

To further examine the effects of age, gender, major, and the subscales of CT on SI in each latent class group, this study conducted regression analyses using the identified potential categories as dependent variables and age, gender, major, and subscales of CT as predictor covariates, with the C1 (low risk of SI) group as the reference group. The results showed (Table 6) that age, gender, major, and SA had no significant effect on the shift in category outcome, whereas EN, PN, EA, and PA had a significant positive effect on the shift to the C2 (high, continuous risk of SI) group (Β = 0.33, 0.32, 0.65, and 0.18, all *P* < 0.01).

**Table 6:** The predictive effects of covariates (C1 as reference)

covariate	*β*	*S.E*	*P*
Age	0.07	0.17	0.674
Gender	-0.11	0.20	0.559
Major	-0.06	0.05	0.146
Emotional Neglect	0.33	0.02	< 0.001
Physical Neglect	0.32	0.04	< 0.001
Emotional Abuse	0.65	0.05	< 0.001
Physical Abuse	0.18	0.05	0.001
Sexual Abuse	0.08	0.08	0.313

Note: C1: “low risk, slowly rising” SI group; C2: “high risk, continuous” SI group; Gender (1 = Male, 2 = Female); Major (1 = Clinical Medicine, 2 = Non-Clinical Medicine)

## Discussion

This is the first study to examine the trajectory of SI among medical students. We investigated SI over a period of one year and used an individual-centered, latent variable, growth mixture modeling to explore the trajectory of SI among medical students to capture some of the developmental patterns of SI over time.

This study assessed CT in medical students, with 30.4% reporting one or more types of abuse and neglect, higher than previous studies [45], possibly due to differences in results using different scales. SI was also assessed before the COVID-19, with a prevalence of 3.9%, lower than the findings of Gao [46] and Bhat [47] for non-medical students and in line with Fan [48] results. Medical students had more opportunities to acquire knowledge about physical and mental health, to master psychological adjustment methods, and to cope positively with frustration, resulting in a lower prevalence of SI. On the other hand, medical students' SI scores and prevalence rates were higher during the pandemic than before. A significant increase in the prevalence of SI among the university student population was commonly reported during the pandemic [49–51].The COVID-19 pandemic had a lasting negative impact, with a range of problems brought about by the epidemic, such as restricted activities, limited social and interpersonal interactions, changes in study styles, and possible financial family economic stress. University students' lack of experience in coping with sudden and serious public health events (e.g. COVID-19) leads to distress and worry, resulting in an increase in SI scores. However, an interesting finding was that the prevalence of SI was lower in the three surveys of this study than in previous studies of health care workers and migraine medical students [52, 53]. That is, the epidemic influenced the occurrence of SI in medical students but did not seriously worsen the prevalence of SI. Following the outbreak, the National Health and Wellness Commission of the People's Republic of China promptly issued guidelines for emergency psychological crisis intervention in pneumonia outbreaks of novel coronavirus infections and quickly established a psychological rescue medical team and a psychological assistance hotline team to carry out psychological crisis interventions for different populations [41]. At the medical school where the subjects of this study were enrolled, they opened the first 24-h psychological crisis intervention hotline in Hainan Province, China, on 30 January 2020 to help people who were in urgent need of emotional relief due to the impact of the epidemic. The WeChat public platform is also used to provide scientific knowledge dissemination, including epidemic prevention and control measures, medical rescue methods, and psychological adjustment methods. These measures can raise awareness about infectious diseases and their ability to regulate their poor psychological state, which is conducive to the prevention of psychological problems [54].

We found heterogeneity in the trajectory of SI development. There were two types of trajectories of SI among medical school students: a low risk, slowly rising group and a high risk, continuous group. First, the most representative group was the low risk, slowly rising group, accounting for 84.5% of the total, a finding that is inconsistent with previous studies [4]. This suggests that the majority of medical students were at a very low initial level of SI that slowly increased over time, meaning that medical students are at increasing risk of suicide over time. This study lasted for 1 year, and the study participants went from the first semester to the third semester of their undergraduate studies. The increased number and difficulty of courses at this more advanced level may have made them feel academically stressed and generated negative emotions such as fear of academic failure, burnout, anxiety and depression, which in turn would make the frequency and level of SI higher [55]. Additionally, at the time of the COVID-19 pandemic, China was on full alert, and the fear among the public and depression among medical students were at their maximum, leading to increased levels of SI. The high risk, continuous group accounted for 15.5% of the total. This group started at a high level of SI, and while over time, these ideas subsided, the decrease was nonsignificant, leaving the group at a high-risk level. This may be due to the lower level of mental resilience, lower levels of feelings of pleasure and lower abilities to cope with adversity when faced with negative events. These combined factors indicate that the individual does not have sufficient intrinsic resources to cope with the impact of negative life events [56], resulting in the level of SI remaining at a high level. When faced with an unexpected public health event, for the general medical student population, mental health lectures and psychological knowledge should be conducted to alleviate panic and anxiety and avoid serious psychological problems, and these professionals conducting the intervention need to be properly resourced and appropriately trained [57]. For specific groups, such as medical students with depression, SI and previous hospitalization history in psychiatric hospitals, we need to focus on using psychological treatment methods such as diagnostic behavior therapy skills training (DBT) and Internet-based cognitive behavior therapy (ICBT) to prevent SI from turning into suicide [58, 59].

The close relationship between emotional neglect, somatic neglect, emotional abuse, physical abuse and SI is consistent with previous studies [60, 61]. Individuals who have been traumatized in childhood live with the stressful expectation that they may be abused at any time in their lives and are prone to higher than usual levels of cortisol arousal; they are insecure about relationships and lack people to turn to for understanding and support when they experience negative events. They are also prone to self-loathing and suicidal thoughts in response to negative events. Emotional neglect, somatic neglect, emotional abuse and physical abuse can affect the development of SI both independently and in combination. This appears to confirm the link between CT and vulnerability to late stress. the experience of CT increases vulnerability to late stressful events, thereby sensitizing individuals to psychopathology [62, 63]. This finding was confirmed during the COVID-19 pandemic [64]. In this study, although the SA had a moderate close relationship with SI at T1, it did not predict SI trajectory class membership. It may be due to group variability in the study population. Most studies of sexual abuse have focused on psychiatric inpatients, those with bipolar disorder and those with depression [65, 66]. These groups all have some impairment in social functioning and poorer interpersonal interactions and are more likely to experience SI than healthy groups.

### Study limitations

We should note that there are some shortcomings in the present study. First, this study has some limitations in terms of data collection, as only students from one medical university in China were tracked, and it is uncertain whether the findings of the study can be generalized to other schools; future studies could validate the results at medical schools in different regions. Secondly, this study did not investigate the prevalence of mental illness among the participants, and future studies could examine whether participants suffered from mental illness, SI co-morbid depression, or physical illness, which could lead to different results. In addition, this study examined only the association between CT and the trajectory of SI, and future research could focus on the association of other influences, such as family functioning, core self-evaluation and school climate, with the trajectory of SI. Future studies could also incorporate endocrine and brain structure findings in individuals with SI. Finally, we conducted only a longitudinal follow-up and did not develop an intervention programme to improve SI. Future research could consider developing targeted programmes for intervention or guidance based on the characteristics of different heterogeneous groups.

## Conclusion

This study identified trajectories of SI among Chinese medical students in a low-level, slowly rising group and a high-level, continuous group. Emotional neglect, somatic neglect, emotional abuse, and somatic abuse can both independently and jointly influence the development of SI. The findings suggest attention should be given to the effects of CT for medical students with high SI.

## Data Availability

The datasets generated during and/or analyzed during the current study are available from the corresponding author on reasonable request.

## References

[1] World Health Organization. WHO guidance to help the world reach the target of reducing suicide rate by 1/3 by 2030. 2021. [https://www.who.int/news/item/17-06-2021-one-in-100-deaths-is-by-suicide]

[2] Klonsky ED, May AM, Saffer BY (2016). Suicide, suicide attempts, and suicidal ideation. Annu Rev Clin Psychol.

[3] Van Orden KA, Cukrowicz KC, Witte TK (2012). Thwarted belongingness and perceived burdensomeness: construct validity and psychometric properties of the Interpersonal Needs questionnaire. Psychol Assess.

[4] Groleger U, Tomori M, Kocmur M (2003). Suicidal ideation in adolescence–an indicator of actual risk?. Isr J Psychiatry Relat Sci.

[5] Eskin M, Sun JM, Abuidhail J (2016). Suicidal behavior and psychological distress in university students: a 12-nation study. Arch Suicide Res.

[6] Yang LS, Zhang ZH, Sun L (2015). Prevalence of suicide attempts among college students in China: a meta-analysis. PLoS One.

[7] Fan AP, Kosik RO, Su TP (2011). Factors associated with suicidal ideation in Taiwanese medical students. Med Teach.

[8] Hill MR, Goicochea S, Merlo LJ (2018). In their own words: stressors facing medical students in the millennial generation. Med Educ Online.

[9] Nan J, Jiang H, Lin L (2022). Latent class and related factors of suicide ideation in medical vocational college freshmen. Chin J Health Psychol.

[10] Van Niekerk L, Scribante L, Raubenheimer PJ (2012). Suicidal ideation and attempt among South African medical students. S Afr Med J.

[11] Kivelä L, Krause-Utz A, Mouthaan J (2019). Longitudinal course of suicidal ideation and predictors of its persistence - A NESDA study. J Affect Disord.

[12] Goldston DB, Erkanli A, Daniel SS (2016). Developmental trajectories of suicidal thoughts and behaviors from adolescence through adulthood. J Am Acad Child Adolesc Psychiatry.

[13] Chen J, Yang J, Zhu Xiong Z (2011). A qualitative-stress model of suicidal ideation in high school students: A multitemporal follow-up study. Psychol Sci.

[14] Xiao Y, Lindsey MA (2021). Adolescent social networks matter for suicidal trajectories: disparities across race/ethnicity, sex, sexual identity, and socioeconomic status. Psychol Med.

[15] Liu Y, Yang Y, Wang CX (2020). The effects of school climate and negative emotions on adolescent suicidal ideation: a cross-lagged study. Psychol Behav Res.

[16] Appleyard K, Egeland B, van Dulmen MH (2005). When more is not better: the role of cumulative risk in child behavior outcomes. J Child Psychol Psychiatry.

[17] Schulz A, Becker M, Van der Auwera S (2014). The impact of childhood trauma on depression: does resilience matter? Population-based results from the study of health in Pomerania. J Psychosom Res.

[18] Bryan CJ, McNaugton-Cassill M, Osman A (2013). The associations of physical and sexual assault with suicide risk in nonclinical military and undergraduate samples. Suicide Life Threat Behav.

[19] Silverman AB, Reinherz HZ, Giaconia RM (1996). The long-term sequelae of child and adolescent abuse: a longitudinal community study. Child Abuse Negl.

[20] Ireland TO, Smith CA, Thornberry TP (2002). Developmental issues in the impact of child maltreatment on later delinquency and drug use. Criminology.

[21] Bahk YC, Jang SK, Choi KH (2017). The relationship between childhood trauma and suicidal ideation: role of maltreatment and potential mediators. Psychiatry Investig.

[22] Kwok SY, Chai W, He X (2013). Child abuse and suicidal ideation among adolescents in China. Child Abuse Negl.

[23] Wilson LC, Newins AR, Kimbrel NA (2019). An examination of the interactive effects of different types of childhood abuse and perceived social support on suicidal ideation. Children’s Health Care.

[24] Zhou Han, Yu S, Liu QX (2019). The relationship between child psychological abuse and neglect and suicidal ideation A mediated model with moderation. Psychol Sci.

[25] Wasserman IM (1992). The impact of epidemic, war, prohibition and media on suicide: United States, 1910–1920. Suicide Life Threat Behav.

[26] Cheung YT, Chau PH, Yip PS (2008). A revisit on older adults suicides and Severe Acute Respiratory Syndrome (SARS) epidemic in Hong Kong. Int J Geriatr Psychiatry.

[27] Tucci V, Moukaddam N, Meadows J (2017). The forgotten plague: psychiatric manifestations of Ebola, Zika, and emerging infectious diseases. J Glob Infect Dis.

[28] Shultz JM, Baingana F, Neria Y (2015). The 2014 Ebola outbreak and mental health: current status and recommended response. JAMA.

[29] Delanerolle G, Zeng Y, Shi JQ (2022). Mental health impact of the Middle East respiratory syndrome, SARS, and COVID-19: A comparative systematic review and meta-analysis. World J Psychiatry.

[30] Gratz KL, Tull MT, Richmond JR (2020). Thwarted belongingness and perceived burdensomeness explain the associations of COVID-19 social and economic consequences to suicide risk. Suicide Life Threat Behav.

[31] Cukrowicz KC, Cheavens JS, Van Orden KA (2011). Perceived burdensomeness and suicide ideation in older adults. Psychol Aging.

[32] Van Orden KA, Witte TK, Cukrowicz KC (2010). The interpersonal theory of suicide. Psychol Rev.

[33] Czeisler MÉ, Lane RI, Petrosky E (2020). Mental health, substance use, and suicidal ideation during the COVID-19 pandemic — United States, June 24–30, 2020. MMWR Morb Mortal Wkly Rep.

[34] Shi L, Que JY, Lu ZA (2021). Prevalence and correlates of suicidal ideation among the general population in China during the COVID-19 pandemic. Eur Psychiatry.

[35] Cheung T, Lam SC, Lee PH (2021). International research collaboration on COVID-19. Global imperative of suicidal ideation in 10 countries amid the COVID-19 pandemic. Front Psychiatry.

[36] Xie M, Tang Y, Zhu L (2021). Childhood trauma and mental health status in general population: a series mediation examination of psychological distress in COVID-19 pandemic and global sleep quality. Front Psychiatry..

[37] Chi X, Becker B, Yu Q (2020). Prevalence and psychosocial correlates of mental health outcomes among Chinese college students during the Coronavirus disease (COVID-19) pandemic. Front Psychiatry.

[38] Tsur N, Abu-Raiya H (2020). COVID-19-related fear and stress among individuals who experienced child abuse: The mediating effect of complex posttraumatic stress disorder. Child Abuse Negl.

[39] Lahav Y (2020). Psychological distress related to COVID-19 - The contribution of continuous traumatic stress. J Affect Disord.

[40] Siegel A, Lahav Y (2022). Emotion regulation and distress during the COVID-19 pandemic: the role of childhood abuse. J Interpers Violence.

[41] Ma N, Ma H, Li LJ (2020). Expert analysis of the guidelines for emergency psychological crisis intervention in pneumonia outbreaks with novel coronavirus infection. Chin J Psychiatry.

[42] Zhaoyun X, Dongbo W, Xudong He, et al. Application of the suicidal ideation self-assessment scale in college students. Chin J Psychiatry. 2007;40(02):94. 10.3760/j.issn:1006-7884.2007.02.015.

[43] He J, Zhong X, Gao Y (2019). Psychometric properties of the Chinese version of the Childhood Trauma Questionnaire-Short Form (CTQ-SF) among undergraduates and depressive patients. Child Abuse Negl.

[44] Ji S, Wang H (2018). A study of the relationship between adverse childhood experiences, life events, and executive function among college students in China. Psicol Reflex Crit.

[45] Lu L, Jian SY, Dong M (2020). Childhood trauma and suicidal ideation among Chinese university students: the mediating effect of Internet addiction and school bullying victimisation. Epidemiol Psychiatr Sci.

[46] Gao T, Xiang YT, Zhang H (2018). Prevalence and correlates of suicidal behaviors among college students in Northeastern China: a cross-sectional study. Psychiatr Q.

[47] Bhat US, Amaresha AC, Kodancha P (2018). Psychological distress among college students of coastal district of Karnataka: A community-based cross-sectional survey. Asian J Psychiatr.

[48] Yong F (2021). The relationship between abuse experience and suicidal ideation among college students in "double-class" universities in the Yangtze River Delta region. Modern Prevent Med.

[49] Liang SW, Liu LL, Peng XD (2022). Prevalence and associated factors of suicidal ideation among college students during the COVID-19 pandemic in China: a 3-wave repeated survey. BMC Psychiatry.

[50] O'Connor RC, Wetherall K, Cleare S (2021). Mental health and well-being during the COVID-19 pandemic: longitudinal analyses of adults in the UK COVID-19 mental health & Wellbeing study. Br J Psychiatry.

[51] Wang D, Ross B, Zhou X (2021). Sleep disturbance predicts suicidal ideation during COVID-19 pandemic: A two-wave longitudinal survey. J Psychiatr Res.

[52] Sahimi HMS, MohdDaud TI, Chan LF (2021). Depression and suicidal ideation in a sample of Malaysian healthcare workers: a preliminary study during the COVID-19 pandemic. Front Psychiatry.

[53] You Aijun, Shi Lu, Shaobo, et al. Suicidal ideation and its related factors among medical college graduate students. Chin Ment Health J. 2020;34(02):147–52. 10.3969/j.issn.1000-6729.2020.2.012.

[54] Khan A, Farooqui A, Guan Y (2015). Lessons to learn from MERS-CoV outbreak in South Korea. J Infect Dev Ctries.

[55] Rigucci S, Sarubbi S, Erbuto D (2021). Negative emotion dysregulation is linked to the intensity of suicidal ideation in a mixed inpatient sample. J Affect Disord.

[56] Wei W, Weili L, Di Z (2016). A follow-up study on suicidal ideation of new recruits during their adaptation period. Chin J Health Psychol.

[57] Knipe D, Padmanathan P, Newton-Howes G (2022). Suicide and self-harm. Lancet.

[58] Lai MH, Maniam T, Chan LF (2014). Caught in the web: a review of web-based suicide prevention. J Med Internet Res.

[59] Chan LF, Shamsul AS, Maniam T (2014). Are predictors of future suicide attempts and the transition from suicidal ideation to suicide attempts shared or distinct: a 12-month prospective study among patients with depressive disorders. Psychiatry Res.

[60] Angelakis I, Gillespie EL, Panagioti M (2019). Childhood maltreatment and adult suicidality: a comprehensive systematic review with meta-analysis. Psychol Med.

[61] Wang KUN-YAN, Lin BING-BING, Ma J-P (2020). Childhood emotional abuse and adolescent suicidal ideation: a mediated model with moderation. Chin J Clin Psychol.

[62] Heim C, Nemeroff CB (2001). The role of childhood trauma in the neurobiology of mood and anxiety disorders: preclinical and clinical studies. Biol Psychiatry.

[63] Koenen KC, Moffitt TE, Poulton R (2007). Early childhood factors associated with the development of post-traumatic stress disorder: results from a longitudinal birth cohort. Psychol Med.

[64] Janiri D, Moccia L, Dattoli L (2021). Emotional dysregulation mediates the impact of childhood trauma on psychological distress: First Italian data during the early phase of COVID-19 outbreak. Aust N Z J Psychiatry.

[65] DeCou CR, Lynch SM (2019). Emotional reactivity, trauma-related distress, and suicidal ideation among adolescent inpatient survivors of sexual abuse. Child Abuse Negl.

[66] Larson RW, Moneta G, Richards MH, Wilson S (2002). Continuity, stability, and change in daily emotional experience across adolescence. Child Dev.

